# The role of poly(ADP-ribose) polymerase inhibitors in the treatment of cancer and methods to overcome resistance: a review

**DOI:** 10.1186/s13578-020-00390-7

**Published:** 2020-03-11

**Authors:** Mausam Patel, Somaira Nowsheen, Sanjay Maraboyina, Fen Xia

**Affiliations:** 1 Department of Radiation Oncology, University of Arkansas for Medical Sciences, 4301 W. Markham St., #771, Little Rock, AR 72205-7199 USA; 2 Mayo Clinic Medical Scientist Training Program, Mayo Clinic Alix School of Medicine and Mayo Clinic Graduate School of Biomedical Sciences, Mayo Clinic, Rochester, MN USA

**Keywords:** PARP, Inhibitors, Review, Resistance

## Abstract

Poly(ADP-ribose) polymerase (PARP) inhibitors represent one of the successful novel approaches to targeted cancer treatment. Indeed, the US Food and Drug Administration (FDA) has recently approved PARP inhibitors for the treatment of breast and ovarian cancers. Despite the proven efficacy of these agents, certain challenges remain with their use. Among the most important are primary and secondary resistance. Here, we review the mechanism of action of PARP inhibitors and their ability to exploit certain inherent deficiencies among malignant cells to improve cell killing, with a focus on deficiencies in homologous recombination among cells with *BRCA1* and *BRCA2* mutations. Moreover, we discuss the different mechanisms of resistance including development of secondary resistance and strategies to overcome them. Finally, we discuss the limitations of novel therapeutic interventions and possible future studies to exploit biochemical pathways in order to improve therapeutic efficacy of PARP inhibitors.

## Introduction

Genomic stability is maintained by intricate and highly complex biochemical pathways regulated by a multitude of proteins. Cells are at risk of mutagenesis and subsequently carcinogenesis when one or more of these pathways is disturbed by biological, physical, or chemical means. Cells attempt to repair damaged DNA through multiple different mechanisms including base excision repair, nucleotide excision repair, mismatch repair, non-homologous end joining (NHEJ), and homologous recombination (HR) pathway [1, 2]. The last two mechanisms target and repair double strand breaks (DSBs) [1].

While genetic aberrations may lead to mutations and malignant transformation, these very aberrations can be exploited to induce targeted cancer cell death [3]. For example, Breast Cancer genes 1 and 2 (BRCA1 and BRAC2, respectively) are necessary for repair of DSBs through the process of HR; and although mutations in these tumor suppressors predispose towards breast and ovarian cancer, these very mutations can be exploited to induce cell death [4, 5]. Poly(ADP-ribose) polymerase (PARP) inhibitors represent one class of targeted therapy that can be used to target cells with BRCA1/2 variants and/or mutations.

**Fig. 1:**
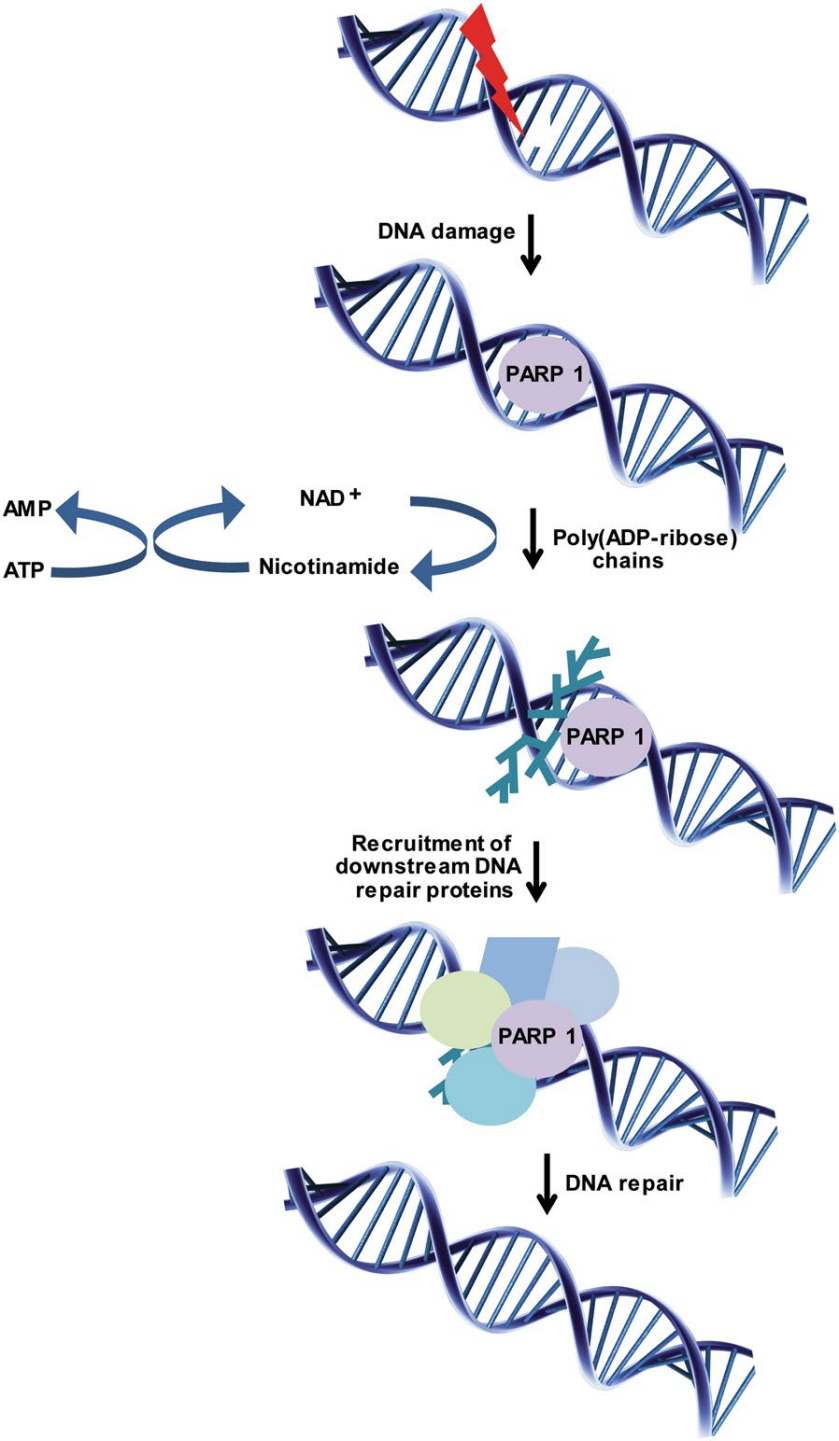
Mechanism of action of poly(ADP-ribose) polymerases in DNA repair

PARPs are enzymes that catalyze the addition of poly(ADP-ribose) (pADPr) adducts to various biological molecules involved in cell signaling and DNA repair. Indeed, PARPs are involved in the recognition and repair of single strand breaks (SSBs). The activation of PARP1 occurs after its N-terminal zinc finger DNA binding domain recognizes and interacts with the SSB. Subsequently, PARP utilizes oxidized nicotinamide adenine dinucleotide (NAD+) as a substrate to catalyze polymers of pADPr. The C-terminal domain then transfers pADPr polymers to both itself and other acceptor proteins. The addition of pADPr adducts leads to the recruitment of hundreds of downstream proteins that regulate DNA repair and eventually repair these SSBs (Fig. 1) [6–8]. However, if these SSBs are not repaired, they eventually progress to DSBs, which are highly cytotoxic to cells.

## PARP family of enzymes

Eighteen PARPs have been described and can be broadly categorized into 3 groups by catalytic activity [9]. PARP1, PARP2, PARP5a and PARP5b catalyze the formation of branching pADPr chains up to 200 units long. PARP9, PARP13, and PARP18 either lack activity or have been uncharacterized. The remaining PARPs only catalyze the addition of a single ADP-ribose unit [mono(ADP-ribose), or mADPr] [9, 10].

Although PARPs involved in the formation of pADPr adducts have been the most well studied and characterized, the majority of PARPs only catalyze the addition of mADPr adducts. PARPs involved in the addition of mADPr have been linked to multiple cellular functions including transcription regulation, signal transduction, unfolded protein response, and actin cytoskeleton/organelle regulation.

Similar to PARPs 1 and 2, PARP10 has been implicated in DNA replication and repair. PARP10 interacts with proliferating cell nuclear antigen (PCNA), a replication fork protein [11]. The association between PARP10 and PCNA helps cells bypass stalls at replication forks and resume DNA synthesis. PARP7, however, has not been directly linked to DNA repair but does function in transcription regulation, cellular autophagy, immunity, and response to viral infections [12]. This review will focus primarily on PARP1 and PARP2 as most inhibitors act upon these enzymes.

## Synthetic lethality

**Fig. 2:**
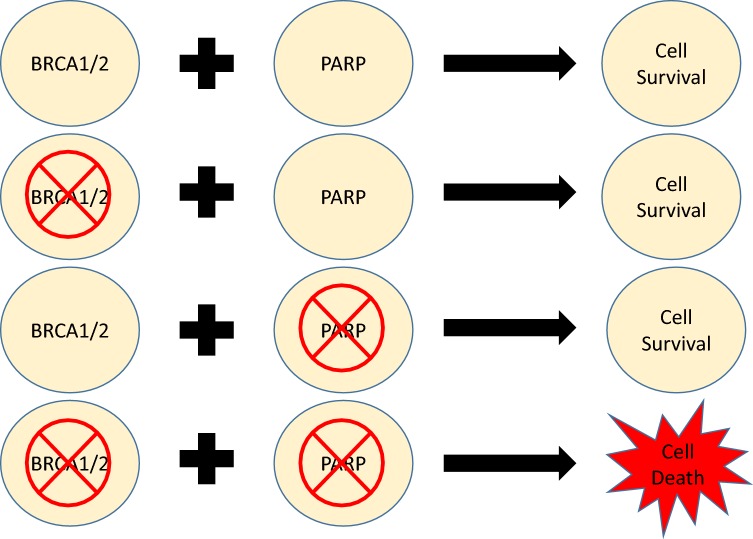
Synthetic lethality demonstrated between BRCA1/2 and PARP. Defects in either BRCA1/2 or PARP does not lead to cell death; however simultaneous defects or inhibition of both proteins leads to cell death

Synthetic lethality is a concept in which a genetic defect in 1 of 2 given genes has no observable effect on the cell but defects in both genes concurrently leads to cell death [13, 14]. Such an interaction between PARP inhibition and BRCA1/2 mutations was first described in 2005 [15, 16]. It has been observed that PARP inhibitors lead to an accumulation of SSBs and stall of replications forks. As these stalled replication forks remain unrepaired, they form DSBs that can be highly lethal to the cell. Logically, tumors lacking the ability to repair DSBs, i.e. those with BRCA1/2 mutations would be expected to be especially sensitive to PARP inhibitors. Indeed, this is the case, as cancer cells with aberrant variants of BRCA1/2 such as breast and ovarian cancers are prone to cell death in the presence of PARP inhibitors, thereby demonstrating synthetic lethality (Fig. 2). Furthermore, mutations in other genes involved in HR including *BLM*, *WRN*, *NBS1*, *FANC*, *CDK12*, and *CHK2* can mimic the mutations of BRCA1/2 and display a similar phenotype. These tumors also demonstrate sensitivity to PARP inhibitors due to their BRCAness, which describes the presence of a deficiency in HR despite wild type BRCA1 and BRCA2 [17].

### Mechanism of action of PARP inhibitors

**Fig. 3:**
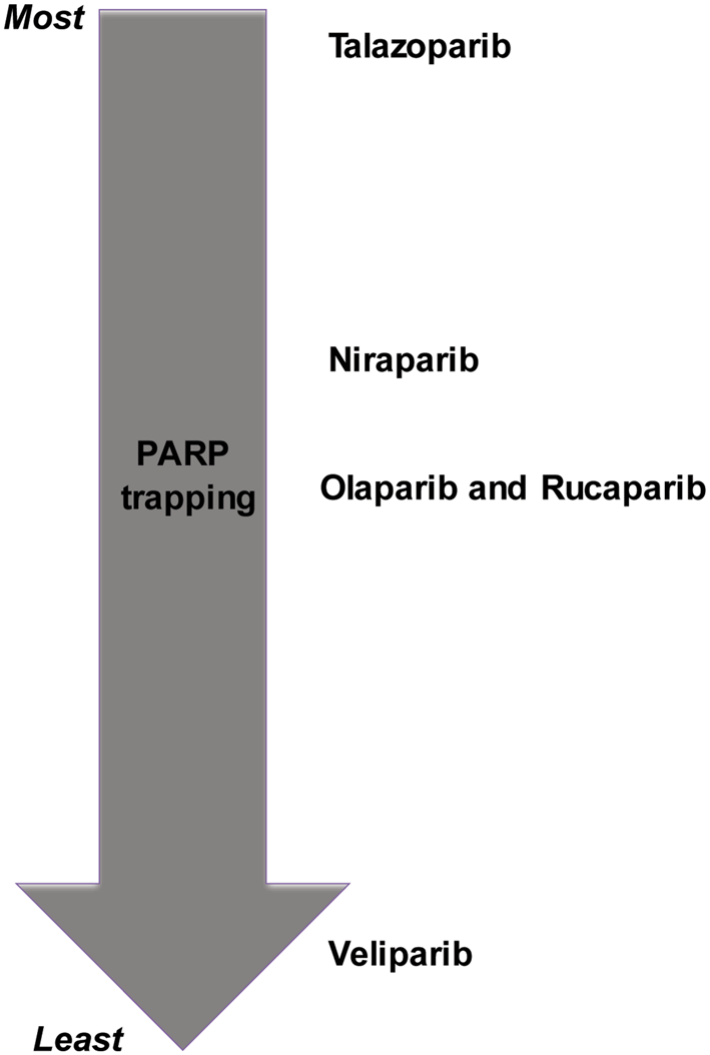
Efficiency of various PARP inhibitors ranked by efficiency of PARP trapping

Multiple PARP inhibitors have been studied including talazoparib, niraparib, olaparib, rucaparib, and veliparib. Several mechanisms explain the efficacy of these agents. Some suppress the catalytic activity of PARPs preventing formation of pADPr chains. This prevents NAD+ from binding to sites of SSBs and activating downstream targets. Other PARP inhibitors occupy the NAD+ binding site and trap PARP1 and PARP2 onto the DNA leading to further polymerization of pADPr. Certainly, PARP trapping is an important concept and is thought to represent a key component of the potency of various PARP inhibitors. It has been suggested that the greater the efficiency of PARP trapping by an inhibitor, the greater the potency [2, 18]. The efficiency of PARP trapping by PARP inhibitor is illustrated in Fig. 3.

## FDA approved PARP inhibitors and significant clinical trials

Thus far, four PARP inhibitors have been approved by the FDA in the U.S. for clinical use including Olaparib, Rucaparib, Niraparib, and Talazoparib [2, 19, 20]. The indications for each FDA approved PARP inhibitor is summarized in Table 1 and described in detail here.

**Table 1:** FDA approved PARP inhibitors for clinical use and associated indications

PARP inhibitor	Clinical indications
Olaparib	Maintenance therapy for platinum sensitive, recurrent, BRCA mutated ovarian cancer after ≥ 3 prior chemo regimens Maintenance therapy for platinum sensitive, recurrent, epithelial ovarian, fallopian, and peritoneal cancer with confirmed or suspected BRCA mutations Treatment of metastatic, HER2 negative, BRCA mutated breast cancer who previously received chemotherapy Treatment of metastatic, BRCA mutated, pancreatic cancer
Rucaparib	Treatment of platinum sensitive, recurrent ovarian, fallopian, and peritoneal cancer
Niraparib	Maintenance therapy for platinum sensitive, recurrent ovarian, fallopian, and peritoneal cancer
Talazoparib	Treatment of BRCA mutated, HER2 negative, advanced breast cancer

### Olaparib

The first PARP inhibitor to be approved, Olaparib (Lynparza, AstraZeneca) was originally approved by the FDA in 2014 for germline mutated BRCA 1/2 ovarian cancer patients who had undergone 3 or more prior regimens of chemotherapy [19]. Various trials demonstrated improved outcomes with Olaparib.

In 2017, based on the results of NCT01874353 and NCT00753545, the FDA approved Olaparib for maintenance treatment of platinum sensitive, recurrent, epithelial ovarian, fallopian, and peritoneal cancer among patients with deleterious or suspected deleterious BRCA mutations [19, 21, 22]. Based on NCT02000622, Olaparib was FDA approved in 2018 for metastatic, human epidermal growth factor receptor type 2 (HER2) negative, BRCA mutated, breast cancer [23].

Finally, and most recently, Olaparib was approved for metastatic, BRCA mutated, pancreatic cancer in 2019 after NCT02184195 demonstrated improved PFS with maintenance Olaparib as compared to placebo [24].

### Rucaparib

The results of NCT01968213 (ARIEL3) led to the accelerated approval of Rucaparib (Rubraca, Clovis Oncology Inc.) in the management of recurrent ovarian, fallopian, and peritoneal cancer in patients with platinum sensitive disease [25, 26]. As with many of the trials assessing the efficacy of Olaparib, PFS was the main endpoint of this phase III randomized trial.

### Niraparib

The FDA approved Niraparib (Zejula, Tesaro) in 2017 for platinum sensitive, recurrent ovarian, fallopian, and peritoneal cancer [2, 19]. NCT01847274 was a randomized, double blind, phase III trial supporting the approval of Niraparib in mutated BRCA tumor patient.

### Talazoparib

In 2018, based upon the results of NCT01945775, Talazoparib (Talzenna, Pfizer Inc.) was FDA approved for BRCA mutated, HER2 negative, advanced breast cancer [2, 19]. Advanced breast cancer included those with locally advanced disease, those not suitable for curative therapy, and those with metastatic disease [27].

## Mechanisms of resistance and strategies to overcome resistance

### Secondary mutations and hypermethylation

**Fig. 4:**
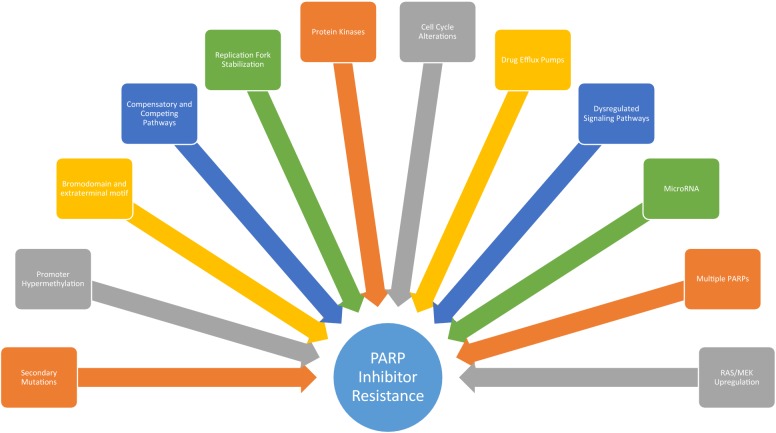
Mechanisms by which tumor cells develop PARP inhibitor resistance

Sustained susceptibility to PARP inhibitors is rare as tumor cells eventually develop resistance. Oftentimes, this occurs due to secondary mutations in genes involved in the HR pathway. For example, secondary mutations in *BRCA1*, *BRCA2, Rad51C* and *Rad51D* can often restore the ability of cells to repair DSBs through HR due to re-institution of in frame gene transcription [28–32]. Moreover, while patients with BRCA1/2 mutations have been the focus of most clinical trials, studies suggest that cells with wild type BRCA1/2 under the control of hyper-methylated promoter regions demonstrate similar sensitivity to PARP inhibitors as those with mutated BRCA1/2 genes [33]. Indeed, inactivation of tumor suppressor genes through epigenetic alterations involving the addition of methyl groups to CpG rich promoter regions has been cited as a common finding among human tumor cells [34]. Such findings have certainly been shown to apply to breast and ovarian cancers with wild type BRCA1/2 genes under the regulation of hypermethylated promoter regions [34, 35]. Thus, tumor cells can also restore HR repair by undoing promoter hypermethylation. This may occur either through active promoter demethylation or through positive selection of pre-existing tumor cells with low levels of BRCA1/2 promoter methylation after PARP inhibitor treatment [36]. Mechanisms of resistance are summarized in Fig. 4.

### Bromodomain and extra-terminal motif (BET) inhibitors

PARP inhibitors tend to be most effective in cells lacking efficient HR given their mechanism of action. Thus, most studies have focused on cell killing in breast and ovarian cancer cell lines, which are more often associated with a lack wild type BRCA1/2. However, a major challenge has been replicating the same proficient cell killing among tumor cells with normal HR function [37–41]. Recent data demonstrates that bromodomain and extra-terminal motif (BET) inhibitors sensitize HR proficient cells to PARP inhibitors inducing a synergistic effect [42–44]. New therapeutic strategies combining PARP and BET inhibitors may thus help overcome not only primary resistance but also the development of secondary resistance.

The action of BET inhibitors appears to be multifactorial. First, they reversibly bind to bromodomains (BRD) of BET proteins blocking interaction with acetylated histones and transcription factors [42]. Inhibition of BET interferes with *BRCA1* and *RAD51* expression, and thus ultimately represses not only wild type, de novo HR but also secondary HR from secondary mutations [42]. Given the simultaneous inhibition of *BRCA1* and *RAD51*, the combination of BET inhibitors with PARP inhibitors may further increase the sensitivity of HR deficient, *BRCA* mutated tumor cells by further downregulating expression of *RAD51*.

Additionally, mitotic catastrophe may be the endpoint for tumor cells with the combination of BET and PARP inhibitors. For instance, the BET inhibitor, JQ1, was shown to decrease levels of the G2-M cell cycle checkpoint regulator, WEE1, and the DNA damage response factor, TOPBP1 [43]. WEE1 functions at the G2-M checkpoint, preventing entry into mitosis in the presence of DNA damage. Additionally, TOPBP1 is associated with the DNA replication and DNA damage signaling pathways. The PARP inhibitor Olaparib represses the functions of both WEE1 and TOPBP1, leading to the accumulation of DNA damage, circumvention of crucial checkpoints, and entrance into mitosis thereby resulting in mitotic catastrophe and cell death [43].

More recent studies have also demonstrated that the inhibition of Bromodomain containing 4, BRD4, a member of the BET protein family, results in the depletion of C-terminal binding protein interacting protein (CtIP). CtIP interacts with MRE11-RAD50-NBS1 (MRN) complex at DSBs activating DNA-end resection, ssDNA formation, and MRN nuclease activity, which ultimately results in DSB repair through HR [44–46]. Therefore, it follows that downregulation of CtIP with BRD4 inhibitors would sensitize normally resistant cells to PARP inhibitors. Indeed, inhibition of BRD4 has been shown to sensitize various tumor cell lineages to PARP inhibitors through the depletion of CtIP and thus, the induction of HR deficiency. These effects have been shown to be independent of *BRCA1/2*, *TP53*, *RAS*, and *BRAF* mutation status [44].

### Compensatory and competing pathways

The repair pathways for DSBs in the cell include HR and NHEJ. There exists a balance between the two pathways with one pathway inhibiting the other. HR and NHEJ are regulated by BRCA1 and 53BP1, respectively [47]. Loss of HR proficiency causes cells to rely on more error prone processes such as NHEJ to repair DNA DSBs. Because the latter is not template based, it leads to joining of DSBs on different chromatids and production of complex chromosomal rearrangements. Interestingly, depletion of 53BP1 in BRCA1 mutated cells has been shown to restore HR activity to near wild type levels [47]. As a result, tumor cells with depletion of 53BP1 gain resistance to PARP inhibitors [47–49]. Mechanistically, 53BP1 binds to chromatid breaks in BRCA1 deficient cells, blocking ATM dependent resection at the site. As ATM dependent resection is necessary for creating single strand breaks required for HR, depletion of 53BP1 ultimately restores ATM dependent HR [47].

REV7, or MAD2L2, is a member of the Shieldin complex, a 53BP1 effector complex [50]. Shieldin is also composed of C20orf196 (SHLD1), FAM35A (SHLD2), and CTC-534A2.2 (SHLD3) [51]. REV7 has been shown to function downstream of 53BP1 to further block DSB resection and thereby HR. This promotes NHEJ. Similar to 53BP1, depletion of REV7 restores HR in BRCA deficient cells. Indeed, depletion of either REV7 or any of the proteins belonging to the Shieldin complex results in resistance to PARP inhibitors in *BRCA1* deficient cells [49, 50]. Moreover, it has been suggested that HSP90 is stabilized in cells with dysfunctional BRCA1 and 53BP1. Thus, HSP90 inhibition may be a potential therapeutic strategy to sensitize *BRCA1* deficient cells with dysfunctional 53BP1 to PARP inhibitors [52].

### Replication fork

The stabilization of replication forks in tumor cells is another pathway by which *BRCA1/2* deficient tumor cells develop resistance to PARP inhibitors [53]. However, this is not due to restoration of HR. Instead, it involves loss of the MLL3/4 protein Pax2 transactivation domain-interacting protein (PTIP) which not only protects *BRCA1/2* deficient cells from DNA damage, but also rescues *BRCA2* deficient cells from death. PTIP deficiency prohibits the recruitment of MRE11 nuclease to replication forks, protecting DNA strands from degradation and stabilizing the replication fork. This ultimately leads to resistance to PARP inhibitors [53]. Given the function of topoisomerases in unwinding the DNA at replication forks, its inhibition should destabilize DNA replication forks. Indeed, the combination of topoisomerase inhibitors and PARP inhibitors have been shown to lead to enhanced cell killing [54, 55]. This represents yet another possible mechanism to overcome PARP inhibitor resistance with multiple phase I/II trials currently underway in patients including NCT02631733; CPT-11, NSC#616,348; and NCT01012817.

### Protein kinases

A-T mutated (ATM) and ATM- and Rad3-related (ATR) proteins are two essential members of the family of phosphoinositide 3-kinase-related kinases (PIKKs) [56]. These two protein kinases play key roles in the DNA damage response pathway regulating hundreds of downstream substrates through phosphorylation [56–58]. This triggers a cascade of events leading to the mobilization of protein repair complexes. Thus, it has been postulated that targeting these important kinases may help overcome resistance to PARP inhibitors. NCT02723864 is a clinical trial evaluating the efficacy of the oral PARP inhibitor, Veliparib, with the ATR inhibitor, VX-970, in combination with cisplatin among patients with refractory solid tumors, including ovarian, esophageal, and NSCLC. Early results from the phase I trial indicate that combination therapy is safe and tolerable [59].

### Cell cycle alterations

Because repair of DSBs through HR is dependent on the presence of a sister chromatid to serve as a template for recombination, the phase of the cell cycle can alter the effectiveness of PARP inhibitors. For example, inhibition of the cyclin dependent kinase 12 (CDK12) transcription regulator with dinaciclib has demonstrated reversal of both de novo and acquired PARP inhibitor resistance [60–63]. CDK12 is a RNA polymerase II C-terminal domain kinase that has been associated with the transcription of various DNA damage response and DNA repair genes including those involved in HR. Given the transcriptional regulation of multiple HR proteins by CDK12; its inhibition represents a promising method to sensitize wild type *BRCA* cells to PARP inhibitors. Furthermore, CDK12 inhibitors have been shown to reverse acquired resistance to PARP inhibitors in both *BRCA* wild type and mutated models of triple negative breast cancer [60].

WEE1 is a tyrosine kinase which regulates cell cycle during the G1-M and S phases through repressive phosphorylation of CDK1 and CDK2, respectively [64]. In the presence of DNA damage, CHK1 phosphorylates WEE1, causing cell cycle arrest to allow for DNA repair [65–67]. The WEE1 inhibitor, AZD1775, has demonstrated efficacy in promoting cell death and apoptosis by sensitizing cells to various chemotherapies including cytarabine, gemcitabine, and cisplatin [68−71].

WEE1 has also demonstrated the ability to inhibit HR mediated repair through activation of CDK1 and inhibitory phosphorylation of BRCA2 [72]. Indeed, the combination of AZD1775 and the PARP inhibitor, olaparib, has been shown to induce apoptosis in acute myeloid leukemia (AML) and acute lymphoblastic leukemia (ALL) cells through the inhibition of HR and subsequent action of olaparib in promoting DSBs [64]. Importantly, the synergistic combination of AZD1775 and olaparib was demonstrated among leukemic cell lines with wild type *BRCA1/2* genes. Moreover, WEE1 inhibition has also been associated with stalled replication forks while PARP has been associated with the recruitment of damage response proteins to stalled forks for processing and repair. Together, these observations indicate that the drug combination of AZD1775 and olaparib may also lead to cell death due to failure of replication fork repair [64, 73, 74].

### Drug efflux pumps

Drug efflux out of the cell is another mode of resistance with upregulation of p-glycoproteins being associated with PARP inhibitor resistance. Upregulation of *Abcb1a/b* genes encoding p-glycoprotein transmembrane efflux pumps has been shown to increase resistance to the PARP inhibitor, AZD2281, in a BRCA1 deficient mammary tumor model [75]. Nonetheless, the resistance was shown to be reversed with the p-glycoprotein inhibitor, tariquidar. Additionally, nanomedicine has been used to evaluate the possible circumvention of resistance to various drugs. Strategies have involved exploitation of endocytosis, using Pluronic nanocarriers, co-deliver of multi-drug resistance (MDR) modulators with chemotherapy, and co-delivery of anti-MDR siRNA with chemotherapy [76–80]. While clinical trials targeting MDR have been unsuccessful in the past, they have not been studied in combination with PARP inhibitors [81]. Additional studies are warranted to determine the role of nanomedicine in overcoming drug efflux pumps in the setting of PARP inhibition.

### Dysregulated signaling pathways

The receptor tyrosine kinase c-Met phosphorylates PARP1 at Tyr907. This leads to activation of PARP1 enzymatic activity and also decreases the ability of PARP inhibitors to bind to their target, thereby conferring PARP inhibitor resistance [82]. However, the combination of c-Met and PARP inhibitors have been shown to overcome this resistance. It has been suggested that an abundance of phosphorylated PARP1 may predict tumor resistance and that the combination of c-Met and PARP inhibitors may benefit patients whose tumors demonstrate high levels of c-Met expression [82].

### MicroRNA

MicroRNAs (miRNAs) are small noncoding RNAs that regulate posttranscriptional genes by either blocking translation of mRNAs by ribosomes or by promoting their degradation [83, 84]. It has been previously shown that the miRNA, miR-182, is involved in repressing BRCA1 protein levels. Blocking the effects of miR-182, as expected, increases BRCA1 protein levels and promotes HR. Thus, antagonizing miR-192 induces resistance to PARP1 inhibitors [85].

In contrast, miR-622 promotes HR through its effect on the Ku complex [86]. It has been demonstrated that miR-622 maintains the balance between HR and NHEJ in *BRCA* deficient ovarian cancer cells. The Ku complex competes with the MRN complex in associating with DSBs with the former diverting the repair pathway to NHEJ and the latter diverting it to HR. Because miR-622 represses the Ku complex during the S phase of the cell cycle, it promotes recruitment of Mre11, which enhances the HR pathway. Therefore, miR-622 leads to PARP inhibitor resistance.

### Targeting other PARPs

There are at least 18 PARP enzymes with different levels of activity [87]. Though PARP1, PARP2, and PARP3 are involved in repair of SSBs, most inhibitors target either PARP1 or PARP2. Therefore, enhancing expression of PARPs other than PARP1/2 may compensate for the inhibitory effects of current PARP inhibitors, ultimately rendering treated tumor cells resistant to conventional PARP inhibition [88, 89]. Thus, in theory, development of PARP inhibitors that target a wide variety of PARP enzymes, such as PARP3, may need to be developed to help circumvent development of resistance. While this approach has not been evaluated at this time, it is likely worth pursuing in the future.

### Combination with immunotherapy

Immunotherapy has long been a topic of great interest and research for the treatment of cancer for several decades [90, 91]. Indeed, the role of immunotherapy in combination with PARP inhibitors has also been explored in the literature [92–95]. One of the major mechanisms by which tumor cells evade the host immune system involves the PD-L1/PD1 pathway. PD-L1 is one of the major inhibitory ligands found on the surface of tumor cells. Interaction of PD-L1 with the PD1 receptor on T cells leads to suppression of T cell proliferation, cytokine release, and cytolytic activity. However, monoclonal antibody blockade of this interaction restores T cell activity and therapeutic anti-tumor activity [96, 97].

PARP inhibition has been demonstrated to upregulate PD-L1 expression, enhancing cancer induced immunosuppression [92]. This indicates that PARP inhibitors may eventually attenuate tumor cell death by promoting host immunosuppression, thus allowing malignant cells to escape T-cell mediated cell death. However, blockade of PD-L1 restores immune mediated anti-tumor activity. Moreover, combined therapy with olaparib and anti-PD-L1 antibody was demonstrated to have greater therapeutic efficacy than either treatment alone in both in vivo and in vitro models [92].

Another potential target for immunotherapy is cytotoxic T-lymphocyte associated protein 4 (CTLA-4). This protein is homologous with the co-stimulatory receptor CD28 and thus, both proteins are able to bind to the same ligands and compete with each other for T cell binding [98]. CD28, in particular, is an immunoglobulin constitutively expressed on most CD4 T cells and about 50% of CD8 T cells. After interacting with its ligand, CD28 enhances signaling pathways that promote T cell proliferation, cytokine secretion, expression of anti-apoptotic genes, and lymphoblast activation. CTLA-4, on the other hand, suppresses T cell activation mainly by outcompeting CD28 for ligand binding.

The combination of PARP inhibitors with CTLA-4 blockade has been explored in the literature, with results demonstrating improved long term survival in a BRCA deficient ovarian tumor model [95]. The enhanced therapeutic effect of combination therapy has been attributed to local increases in interferon γ (IFNγ) by T cells within the tumor environment. Not only did combination therapy demonstrated rapid increases in T cell recruitment, activation, and cytokine secretion; but it also led to induction of long term systemic effector/memory T cell immunity.

Based on these results, a 2-phase model has been suggested to explain the therapeutic effect of PARP inhibition with anti-CTLA-4 antibodies. During phase I, PARP inhibitors directly induce tumor cell damage. This activates an anti-tumor T cell response, which is further amplified by CTLA-4 blockade [99]. In phase II, the activated T cells produce IFNγ above the normal threshold required to enhance the cytotoxicity of PARP inhibition. Thus, the combination of the 2 treatment modalities amplifies the cytotoxicity of PARP inhibitors in the presence of CTLA-4 blockade [95].

### Combination with traditional chemotherapeutic agents

Interestingly, PARP inhibition can also sensitize typically resistance tumor cells to genotoxic chemotherapy [19]. Temozolomide (TMZ) is a DNA methylating drug used for the treatment of gliomas that has demonstrated efficacy with the combination of PARP inhibitors in animal models [100–104]. Moreover, PARP inhibition has been shown to reverse TMZ resistance in tumor models. TMZ adds methyl units to multiple locations in DNA but its cytotoxicity has been attributed to methylation of O^6^ of guanine, leading to base mismatch, futile attempts at repair, DSBs, cessation of growth, and cellular apoptosis [103]. Resistance to TMZ occurs in one of two ways: (1) the DNA repair protein, O^6^-alkylguanine-DNA alkyltransferase (AGT) can directly remove the methyl group at O^6^ and (2) base excision repair (BER) at other methylated sites such as N^3^ of adenine and N^7^ of guanine can restore DNA integrity and allow cells to continue proliferation. PARP inhibition, thus, prevents tumor cells from undergoing BER and repairing DSBs ultimately re-sensitizing them to TMZ [19, 103].

Platinum based chemotherapy acts by covalent bonding to DNA resulting in the formation of DNA adducts and intra-strand cross links [19, 105]. These cross-links interfere with replication and transcription and ultimately lead to apoptosis. Because DNA repair is one of the mechanisms by which tumor cells develop resistance to platinum-based compounds, PARP inhibition has been hypothesized to re-sensitize resistance cells. The efficacy of platinum with PARP inhibitors has also been supported by clinical trials, which have demonstrated improved pathological complete response in triple negative breast cancer and improved PFS in ovarian cancer [106, 107].

### Other targeted therapy

Synthetic lethality has been demonstrated with combination of androgen deprivation therapy (ADT) and PARP inhibitors in prostate cancer [108, 109]. The androgen receptor (AR) is a ligand activated transcription factor involved in the progression of prostate cancer. Moreover, AR signaling has been linked to the activation of HR through the accumulation of γH2AX and RAD51 foci in DNA. Blockade of AR signaling with ADT upregulates PARP activity and is necessary for prostate cancer cell survival. Thus, the combination of ADT and PARP inhibitors has been suggested to be a novel approach to the treatment in prostate cancer. Indeed, the clinical efficacy has also been demonstrated in a phase II, randomized clinical trial which demonstrated improved median PFS with combination therapy as compared to ADT alone [110].

Similarly, the combination of mitogen-activated protein kinase (MEK) inhibitors with PARP inhibitors has also demonstrated synergy in producing cytotoxicity in mutant RAS tumor cell lines [111]. Moreover, cytotoxicity may be independent of *BRCA 1/2* and *p53* mutation status. Resistance to PARP inhibitors has been associated with increased levels of RAS/MEK. MEK and PARP inhibition in combination induce apoptosis in both RAS mutant cell lines and otherwise PARP inhibitor resistant cell lines. These observations have been attributed to alterations in apoptotic balance, induction of HR deficiency, and downregulation of DNA checkpoint function [111].

## Limitations and future directions

As with any therapy, the potential toxicities of PARP inhibitors either alone or in combination with other agents needs to be considered. Systemic therapy is toxic and can affect both normal and cancer cells. At the patient level, drug side effects need to be considered. Oftentimes, nausea, vomiting, immunosuppression, and myelosuppression can be serious adverse effects that need to be taken into account. Indeed, there are multiple phase I/II trials assessing these effects in the context of PARP inhibitors and other systemic agents.

Another limitation in the treatment of tumors is the fast rate at which they grow and the heterogeneous cells that exist within the tumor. Knowledge of DNA damage response pathways and mutational profiles of resistant tumors should help improve outcomes. There has been recent interest in studying the mutational molecular and genetic profile of various tumors [112, 113]. Efforts such as these will help improve targeted therapy and improve current strategies to overcome tumor cell resistance.

The identification of patients who will benefit most from PARP inhibitors will also be important to help maximize therapeutic efficacy and minimize unnecessary drug side effects among patients who would not benefit from PARP inhibition therapy. For instance, immunohistochemistry for BRCA1 and p53 have demonstrated use as surrogate markers to predict resistance and response [114–117]. Data from ongoing clinical trials will help elucidate the best biomarkers for predicting PARP inhibitor response among patients.

## Conclusions

PARP inhibitors have demonstrated efficacy against certain, carefully selected cell lines, typically those with *BRCA1* and *BRCA2* mutations. As such, challenges remain when using such targeted therapy. The most obvious is whether PARP inhibitors can be extended to malignant cells, which harbor wild type *BRCA*1/2 genes. Here, we have reviewed multiple possible mechanisms to overcome primary resistance including concurrent use of BET and WEE1 inhibitors. Additionally, exploitation of miRNAs that regulate *BRCA* protein expression may represent a possible future therapeutic strategy to overcome primary resistance.

Even among sensitive cell lines, the development of secondary resistance remains an obstacle with prolonged use of PARP inhibitors. Indeed, most tumors eventually develop resistance through several different mechanisms. Thus, novel approaches to circumvent secondary resistance need to be further studied. Depending on the mechanism of resistance, multiple approaches have been evaluated to re-established sensitivity to PARP inhibition. These include inhibition of HSP90, CDK12, drug efflux pumps, c-Met, and MEK. Moreover, targeting multiple PARPs and protein kinases, and combining PARP inhibition with immunotherapy/chemotherapy represent other potential strategies to increase therapeutic efficacy.

One benefit of many of the novel approaches described here is that they target multiple pathways. Given the heterogeneous nature of tumors, new therapeutic interventions will need to target tumors from multiple fronts, with the hopes of increasing therapeutic efficacy. Moreover, this will help prevent secondary selection of resistant tumor cells. While much remains unknown, multiple pre-clinical and clinical studies are already underway to not only help establish the role of PARP inhibitors in the treatment of cancer but also determine the optimal approach to overcome resistance.

## Data Availability

Not applicable.
